# Optimized Extraction of Resveratrol from* Arachis repens* Handro by Ultrasound and Microwave: A Correlation Study with the Antioxidant Properties and Phenol Contents

**DOI:** 10.1155/2016/5890897

**Published:** 2016-12-27

**Authors:** Leonardo Garcia, Renata Garcia, Georgia Pacheco, Felipe Sutili, Rodrigo De Souza, Elisabeth Mansur, Ivana Leal

**Affiliations:** ^1^Laboratório de Micropropagação e Transformação de Plantas, Cellular Biology Department, State University of Rio de Janeiro, Rio de Janeiro, RJ, Brazil; ^2^Departamento de Engenharia de Bioprocessos e Biotecnologia, Universidade Estadual Paulista (UNESP), Campus Botucatu, 18610 307 Botucatu, SP, Brazil; ^3^Biocatalysis and Organic Synthesis Group, Chemistry Institute, Federal University of Rio de Janeiro, 22941-909 Rio de Janeiro, RJ, Brazil; ^4^Laboratório de Produtos Naturais e Ensaios Biológicos (LaProNEB), Pharmacy Faculty, Department of Natural Products and Food, Federal University of Rio de Janeiro, 21.941-902 Rio de Janeiro, RJ, Brazil

## Abstract

The vegetal species* Arachis repens*, commonly known as peanut grass, was studied and, for the first time, we detected the presence of the bioactive compound* trans-*resveratrol (*t*-RSV). We compared the efficiency of three different methodologies (conventional maceration [CM], ultrasound-assisted extractions [UAE], and microwave-assisted extractions [MAE]) concerning total phenolics (TP) and resveratrol (*t*-RSV) content, followed by antioxidant activity (AA) evaluation. By CM, at 1 h, the highest RSV content (1.024 ± 0.036 mg/L) and, correspondingly, the highest DPPH capture (23.90 ± 0.04%) were found. The TP contents, at 1 h, presented the highest value (27.26 ± 0.26 mg/g GAE). By the UAE, the maximum yields of TP (357.18 mg/g GAE) and RSV (2.14 mg/L), as well as, the highest AA (70.95%), were obtained by 5 min after a maceration pretreatment, on the solid-solvent ratio 1 : 40 w/v. For MAE, a central composite rotatable design (CCRD) was applied followed by the FFD design in order to evaluate the statistical effects of four independent variables on the extraction of RSV. The optimal conditions established for obtaining the highest recovery (2.516 mg/g) were 20 min; 90% MeOH aq.; 120 rpm; 60°C; and solid-solvent ratio: 1 : 35 w/v. Relevant correlations were established considering the TP and RSV contents, as well as the AA, corroborating obvious advantages of such techniques in terms of high extraction efficiency in shorter times.

## 1. Introduction

The genus* Arachis* L. (Fabaceae) is native from the South America and descriptions suggest that it might be original from Brazil [1]. The genus has more than 80 species already described, grouped into nine taxonomic sections. The most economically important species of the genus is* Arachis hypogaea* L., the fourth oleaginous plant consumed in the world [1, 2]. In addition to* A. hypogaea*, other species are also used for alimentary consumption, ornamental, and weed control uses [1]. The species* Arachis repens*, for example, commonly known as peanut grass, is used as ornamental, forage, and ground cover, in substitution to common grass species. Different bioactive substances have already been identified in* Arachis* species, including the bioactive compound resveratrol, which is abundantly found in the skin of grapes [3]. Resveratrol (3,5,4-trihydroxy stilbene) is a polyphenol found in a variety of vegetal species, such as lilies, mulberries, eucalyptus, pines, peanuts, and grapevine [4]. It is generally synthesized as a defense response to stress, such as UV irradiation, microbial infection, and mechanical damage. Over the past three decades, resveratrol has been receiving especial attention because of its associated health benefits [5], which include positive therapeutic effects on cardiovascular and neurodegenerative diseases, cancer, and inflammation [6–8], in addition to antioxidant and inhibition of platelet aggregation activities [9–11].

The extractive methodologies and, consequently, the solvents employed can be very critical for final compound quality, conducted to additional purification steps. In the case of* trans*-resveratrol, it is susceptible to photochemical isomerization [12]. Thus, a method for the extraction of* trans*-resveratrol from peanut has to include protective measurements such as absence of light, use of inert atmosphere, or addition of an antioxidant with higher antioxidant ability than* trans*-resveratrol [13]. In recent years, the development and use of environmentally friendly extractive methods has become increasingly popular since they can generate products with higher yields and with best quality. In this context, Piñeiro and colleagues [14], on their study based on the efficiency of extraction methods from grapes, reported that the use of ultrasound-assisted extraction (UAE), by 15 min, was effective for the extraction of piceatannol,* trans*-resveratrol, viniferin, and vitisin-B. The efficiency of ultrasound is explained by the higher simultaneous hydration and fragmentation process, while it facilitates mass transference of solutes by the solvent without significant decomposition [15]. It is important to highlight that the chemical and physical effects of ultrasound arise from the cavitational collapse, which give rise to severe conditions and, thus, induce the extraction, not so easily attained under other conventional conditions [16]. Another technology that has been recognized for presenting various advantages over conventional extraction methods is the microwave-assisted extraction (MAE). The MAE has already been applied in a variety of scientific areas; however, the interest focusing on the secondary metabolite extraction has significantly increased over recent years [12]. Conventional extraction methods such as maceration, soxhlet, and solid phase have been associated with high solvent requirements, longer extraction times, and increased risk of thermolabile constituent's degradation. In MAE, the solvent and sample are contained in sealed extraction vessels under controlled temperature and pressure conditions. The closed vessels allow the temperature of the solvent to rise well above its boiling point, with shorter extraction times. Considering that, MAE and UAE offer higher reproducibility, lower solvent consumption, temperature, and power input [17], and, subsequently, increase extraction efficiency [18].

The purpose of this work was to compare the efficiency of three different extractive methodologies in optimized conditions (conventional maceration, ultrasound, and microwave) on the extraction of* trans*-resveratrol, a bioactive constituent detected for the first time in the leaves of* A. repens*. Afterwards, we evaluated the antioxidant activity of the different extracts and the total phenolics and* trans*-resveratrol contents, establishing a correlation between these outcomes. In addition, a fractional factorial design (FFD) followed by a central composite rotatable design (CCRD) was applied for the MAE experiments in order to evaluate the effects of four independent variables, as follows: stirring (900–1200 rpm), temperature (30–60°C), solvent (70–90% methanol), and solvent : mass ratio (1 : 35–1 : 15 w/v) on the extraction yield of* trans*-resveratrol.

## 2. Materials and Methods

### 2.1. Plant Material

The vegetal species* Arachis repens* was commercially purchased on the Municipal Market from Rio de Janeiro (CADEG) in 03/2012. The documentary herbarium specimens were identified by Ph.D. José Francisco Valls (EMBRAPA) and deposited at the Herbarium of the State University of Rio de Janeiro (HRJ-11767). The plants were cultured under greenhouse conditions in pots containing Plantmax® (25 ± 2°C temperature, luminosity measured by each 12 h/12 h; and light intensity in a clear day during the growing period was as high as 1600 *μ*E/(m^2^/s)). Posteriorly (after 30 days), peanut leaves were collected and dried (TECNAL® TE-393/1-MP) at 45°C for 24 h and stored at −20°C until experimental analysis.

### 2.2. Extractive Methodologies

#### 2.2.1. Conventional Maceration Extraction (CME)

In order to determine the optimal length of time for total phenolic compounds and* trans*-resveratrol extraction by using the Conventional Maceration Extraction (CME), dried materials (g) were macerated in a rate of 0.1 g per 4 mL of methanol 80% at room temperature for 30 minutes and 1, 2, and 4 hours. The extractive solutions were then filtered by paper filter and evaporated until dryness in a rotatory evaporator under vacuum (MARCONI®, M120) at 45°C.

#### 2.2.2. Ultrasound-Assisted Extraction (UAE)

In order to determine the optimal conditions by the ultrasound-assisted extraction (UAE) method, the samples were suspended in amber vials (10 mL) at different solid/liquid ratios (1 : 20; 1 : 30; 1 : 40; 1 : 50; and 1 : 60) and methanol concentrations (40, 60, 80, and 100%) (MeOH aq.). The jars were placed in a sonication tank 3/4 filled with water. The suspensions for each combination were sonicated for 5, 10, 20, and 30 minutes. In addition to these parameters, the influence of the maceration before treatment by different times (5, 10, 20, and 30 minutes) was also evaluated. The UAE was performed in an ultrasonic bath (UNIQUE®) with 120 W power and 40 kHz frequency. The equipment consisted of a rectangular tank (0.30 × 0.15 × 0.10 m) with a useful volume of 3.8 L and a digital panel for time settings. The temperature was monitored by each 5 minutes. Subsequently, the extracts were filtered and the filtrates were evaporated until dryness under vacuum in a rotatory evaporator. Then, the extracts were weighted and stored at 4°C until subsequent analysis. The experiments were carried out in triplicate.

#### 2.2.3. Microwave-Assisted Extraction (MAE)

In order to determine the microwave-assisted extraction (MAE) optimal conditions for* trans*-resveratrol extraction, experiments were performed in an Anton Paar® brand microwave apparatus (Monowave™ 300). The effects of the temperature (30–60°C), solvent ratio (70–90% methanol-water) (v/v), stirring (900–1200 rpm), and mass proportion (solid-solvent) (1 : 15–1 : 35 w/v) were evaluated. The extractions were performed at fixed pressure (6 bars) and time (20 min). The microwave irradiation equipment was operated in a temperature control mode, internally measured by a ruby thermometer. The obtained materials were then filtered, evaporated, and stored at 4°C.

### 2.3. Determination of Total Phenolic Content (TPC)

The contents of phenolic compounds in the extracts were measured by the Folin-Ciocalteu method according to Holland et al. [19]. For each sample, 180 *μ*L of Folin-Ciocalteu reagent (Sigma-Aldrich®, Milwaukee, Wisconsin, USA) at 10% (v/v) was added to 90 *μ*L of the tested extract (10 mg/mL) in 96-well microplates. After 5 minutes, 730 *μ*L of Na_2_CO_3_ (100 mM) was added, followed by incubation in the dark for 1 h. After this period, the absorbance was measured at 765 nm using a spectrophotometer (Thermo Scientific, UV-Visible Spectrophotometer, Biomate 3 s). The total phenolic contents were calculated using the calibration curve of gallic acid plotted in five different concentrations and expressed in terms of gallic acid equivalents (GAE).

### 2.4. Determination of the Antioxidant Activity by the DPPH Method

The radical-scavenging activity (RSA) of the extracts was determined based on the methodology described by Brand-Williams et al. [20] by using the 1,1-diphenyl-2-picrylhydrazyl free radical (DPPH) (Sigma-Aldrich). This activity was determined aiming to evaluate the ability of the samples to neutralize the free radical by reducing it. Briefly, samples of the plant extracts at 10 mg/mL diluted in methanol (25 *μ*L) were added to the DPPH solution (60 *μ*M) (975 *μ*L) in 96-well microplates. The mixture (total volume of 1.0 mL) was shaken vigorously and allowed to react at 25°C for 60 min. After this period, the absorbance was measured at 515 nm on a Thermo Scientific, UV-Visible Spectrophotometer, using absolute methanol as blank control. For preparation of the standard curve, different concentrations of DPPH methanol solutions (0–60 *μ*g/mL) were used. The radical-scavenging activity percentage was calculated using the following formula: % RSA = [1 − (absorbance of DPPH – absorbance of sample)/absorbance of DPPH] × 100. All determinations were performed in triplicate.

### 2.5. HPLC-DAD Analysis for the Quantification of* trans*-Resveratrol

Aliquots of the extracts (10 mg/mL) were filtered through a nylon filter (45 *μ*m) prior to HPLC-DAD analysis. Quantification of *t*-resveratrol (in *μ*g/mL) was performed by plotting the results into a calibration curve constructed with a reference pure standard in five different concentrations (1.0; 2.5; 5.0; 10.0; and 20.0 mg/L) (*R*^2^ = 0.9996). The analyses were performed by using a C18 column (Dionex Bonded Silica Products, 5 *μ*m, 120 Å, 4.6 × 250 mm) on a HPLC-DAD (Ultimate 3000 Liquid Chromatography; Dionex Co., São Paulo, Brazil) operated at 307 nm. The sample injection volume was 20 *μ*L, and the gradient elution protocol was performed with three solvents (A: ultrapure water, B: methanol, and C: acetonitrile) with a flow rate of 1 mL·min^−1^. Organic solvents were purchased from Tedia®. The elution profile was established as follows: 0–2 min, 90% solvent A, 8% solvent B, and 2% solvent C; linear gradient from 90 to 70% solvent A, 2 to 22% solvent C; 2–10 min, linear gradient from 70 to 5% solvent A, 22–95% solvent C; 10–18 min, washing with 100% solvent C. The identification and quantification of *t*-resveratrol were carried out by comparison of the retention times and peak areas, with those of *t*-resveratrol standard or by coinjection with the sample (spike test), respectively.

### 2.6. Statistical Analysis

Statistical analyses of maceration and ultrasound-assisted extraction results were conducted with the GraphPad InStat of replicate test data. Analysis of variance was performed by ANOVA and data were reported as mean ± standard error. The experimental designs and results analysis of FFD and CCRD were carried out using the* software* Statistica 6.0 (StatSoft, Inc., USA), according to the significance level established to obtain the mathematical model. The significance of the regression coefficients and the associated probabilities, *p*(*t*), was determined by Student's *t*-test; the model equation significance was determined by Fisher's *F* test. The variance explained by the model is given by the multiple determination coefficients, *R*^2^.

## 3. Results and Discussions

### 3.1. Maceration Method

**Figure 1:**
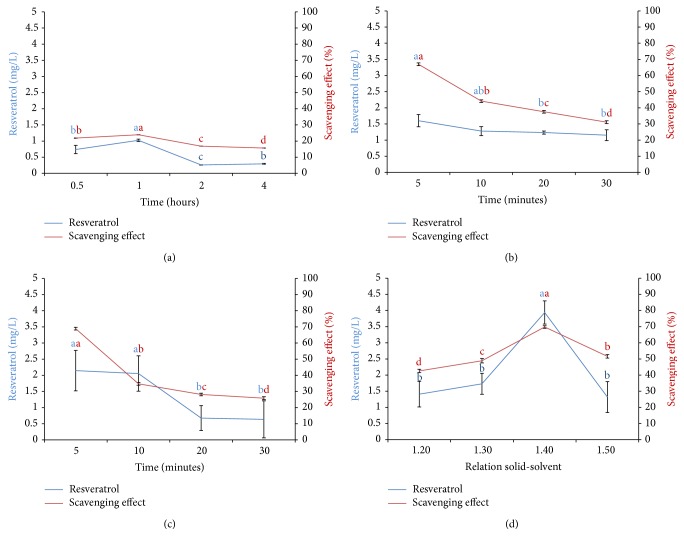
(a) Effect of the time exposure efficiency on the *t*-resveratrol contents in extracts obtained by maceration with 80% methanol (0.1 g per 4 mL), correlated to the radical-scavenging activity. (b) Effect of the resveratrol contents in extracts obtained by ultrasound with 80% methanol (0.1 g per 4 mL) correlated to the radical-scavenging activity. (c) Effect of the time efficiency maceration before treatment on the resveratrol contents in extracts obtained by maceration with 80% methanol (0.1 g per 4 mL) previously exposed to ultrasound for 5 minutes, correlated to the radical-scavenging activity. (d) Correlation of resveratrol contents in extracts at different proportions of solid-solvent, obtained by ultrasound, correlated to the radical-scavenging activity. Means ± standard error followed by the same letter are not statistically different at *p* = 0.05, Tukey-Kramer test.

The effect of the time on the extraction of* trans*-resveratrol adopting 80% MeOH aq. (v/v) as solvent system, and its correlation with the DPPH scavenging activity was established (Figure 1(a)). It was found that in longer times (>1 h) the content of* trans*-resveratrol is decreased, and it is observed as a reducing percentage on the antioxidant activity. All results presented statistical difference and the time of 1 h exhibited the highest percentage of capture of DPPH (23.90 ± 0.04%) and* trans*-resveratrol content (1.024 ± 0.036 mg/L). In addition, the total phenolic compounds contents obtained from the peanut, at different extraction times, revealed that the extract prepared in methanol (80% MeOH aq.) (v/v) with 1 h of exposure to maceration also exhibited the highest content (58.10 ± 1.36 mg GAE) (Table 1) compared to the others. Thus, this time was selected for subsequent analyses.

### 3.2. Ultrasound-Assisted Extraction (UAE)

#### 3.2.1. Optimization of Extraction Time

Figure 1(b) shows the correlation between the* trans*-resveratrol concentration and the DPPH capture of the extracts (80% MeOH aq.) (0.1 g per 4 mL) exposed to ultrasound in different times (5 to 30 minutes). It can be shown that 5 minutes of exposure to ultrasound provided the highest amount of* trans*-resveratrol contents (1.60 ± 0.18 mg/L) compared to 20 and 30 minutes and, correspondingly, the highest percentage of DPPH capture (66.98 ± 0.85%).

Therefore, no statistical differences between 10, 20, and 30 min in terms of* trans*-resveratrol concentration were observed; in contrast, the scavenging activity is modified over the time, suggesting no direct correlation between these parameters on the conditions evaluated. Hence, the shortest period was selected for the extraction time in further analysis. This result corroborates with the speed effect of the ultrasound irradiation on the extraction process.

#### 3.2.2. Optimization of the Pretreatment Time

Samples from the leaf extracts of* A. repens* were macerated on different times (before treatments) and, then, sonicated for 5 min. A pretreatment carried out for 5 min, in 80% methanol, was considered ideal because it is conducted to the highest content of* trans*-resveratrol (2.14 ± 0.62 mg/L) and percentage of DPPH scavenging (70.95 ± 0.83%) (Figure 1(c)). Both parameters were negatively influenced by longer pretreatment exposure times, with significant statistical differences (*p* < 0.05), exceptionally, in terms of* trans*-resveratrol concentration after 5 and 10 min, which were considered identical. Thus, we fixed 5 min for the following experiments.

#### 3.2.3. Optimization of the Solid-Solvent Ratio

A series of extractions was performed in order to verify the ideal solid-liquid ratios concerning the* trans*-resveratrol extraction. The 1 : 40 (solid-liquid) presented the highest content of* trans*-resveratrol (3.93 ± 0.35 mg/L). Thus, this ratio was considered suitable for the extraction. The proportion of 0.1 g/4 mL was also sufficient to provide greater capture of DPPH (66.98 ± 0.85%) (Figure 1(d)).

#### 3.2.4. Determination of Total Phenols Content (TPC)

The results obtained concerning the determination of total phenols content (TPC), expressed as GAE/g (*y* = 0.0101*X* + 0.0216; *R*^2^ = 0.997) of the extract, were observed in the range of 140.82 ± 1.13 to 212.51 ± 2.22 mg GAE g^−1^ in extracts exposed to 30 min and 5 min of ultrasound irradiation, respectively (Table 1). The TPP contents were further evaluated in methanol extracts obtained from different maceration pretreatment times (followed by 5 min ultrasound), and a range between 104.14 ± 1.52 (30 min) and 357.18 ± 1.83 (5 min) mg GAE g^−1^ was observed. These data corroborate the importance of shortest times concerning TPP contents. All results presented statistical differences and the analysis revealed that the extract prepared by 5 min on ultrasound exposure, in proportion of 40 mL/g of material, exhibited the highest phenolic contents (Table 1). These results are in accordance with the DPPH scavenging percentage, since both decrease over the time, suggesting that the phenol compounds must be responsible for the antioxidant activity observed. This data can also be correlated to the highest content of* trans*-resveratrol (2.14 ± 0.62 mg/L) and percentage of DPPH scavenging activity (70.95 ± 0.83%) at this same time. The TPC values in the range of 246.83–356.17 mg GAE g^−1^ were observed for extracts with different solid-solvent ratios, in response to exposure to ultrasound for 5 min, after a pretreatment by maceration for 5 min. In general, the presence of higher content of phenolic compounds on the extracts is correlated to increased antioxidant activity, as can be seen in Table 1 versus Figure 1 (entry 2 versus Figure 1(a); entry 5 versus Figure 1(b); entry 9 versus Figure 1(c); entry 15 versus Figure 1(d)) suggesting relevant direct correlations.

It is important to highlight that the maceration process required longer times for extracting minor amounts of total phenolic compounds (Table 1; entries 1 to 4) and* trans*-resveratrol (Figure 1(a)) compared to the ultrasound-assisted extraction (Table 1; entries 5 to 8) (Figure 1(b)). In addition, macerated extracts lead to reduced antioxidant activity (Figure 1(a)), in comparison to the ultrasound-assisted extraction (Figure 1(b)). In brief, the maximum yield of total phenolic compounds extracted by the ultrasound-assisted extraction (357.18 ± 1.83 mg GAE g^−1^) was in response to exposure to ultrasound for 5 minutes after a pretreatment by maceration for 5 minutes, with 1.40 (w/v) solid-solvent ratio (Table 1).

Within the evaluated parameters, the use of ultrasound equipment showed to have higher efficiency than the conventional maceration method that has already been used in previous works.

### 3.3. Microwave-Assisted Extraction

#### 3.3.1. Initial Evaluation of Reaction Parameters Using Fractional Factorial Design (FFD)

Microwave-assisted extraction consists of treating the sample in an organic solvent (extractant) with microwave energy. The partitioning of the analytes from the sample matrix to the extractor depends on the temperature and the polarity of the solvent. The highly localized temperature and pressure due to volumetric heating of residual moisture present in the plant cell cause selective migration of target compounds from the material to the surroundings. It occurs in a more rapid rate and with similar or better recoveries compared to conventional extractions. In order to optimize the reaction conditions for the extraction of* trans*-resveratrol we have proposed a preliminary study based on a fractional factorial design (FFD), in a two-level model. Initially, we carried out a fractional factorial design 2^4–1^ in order to determine the variables that most influenced the extraction process. This first part of the study required 11 experiments. The variables studied on the FFD were temperature (*T*), solvent : mass proportion (*M*), solvent composition (*S*), and stirring (St). The reaction time was not considered as a variable in the present experimental design, since a kinetic study was previously held in order to establish the best time for the* trans*-resveratrol extraction. The variables with the respective real and coded values attributed and the experimental design with the corresponding results are shown in Table 2. As shown in Table 2,* trans*-resveratrol presented different concentrations (ranging from 0.085 to 0.786 mg/g), depending on the values of the variables applied. In two experiments, it is possible to observe a significant concentration of* trans*-resveratrol (entries 2 and 4). The best result (0.786 mg/g) was achieved at 1200 rpm; 30°C; 60% MeOH aq. solvent; and solvent : mass proportion 1 : 20 (entry 2). Based on the estimated effects for the variables investigated (Supplemental Material 1A available online at http://dx.doi.org/10.1155/2016/5890897) it is possible to see that the temperature (−0.103) and the amount of solvent (−0.211) presented negative effects within the range studied. In contrast, stirring and solvent : mass proportion presented relevant positive effects (0.243 and 0.125, resp.), which are completely in agreement with the conditions described in entry 2 for the best* trans*-resveratrol extraction. All parameters studied showed statistical significance in the process (*p* < 0.05) and the need for a curvature in the mathematical model by including axial points was observed, since it presented *p* < 0.05. Thus, a central composite rotatable design (CCRD) model was further applied.

#### 3.3.2. Central Composite Rotatable Design (CCRD)

In this part of the work we optimized a batch process for the extraction of* trans*-resveratrol by using the response surface methodology (RSM) in a laboratory setting [21]. The RSM has been employed as a statistical tool for developing and optimizing reaction conditions in order to maximize the yields of target products, influenced by several variables [22]. The advantage of RSM is that it allows the user to gather large amounts of information from a small number of experiments [23] and to observe the effects of individual variables and their combination on the response.

**Figure 2:**
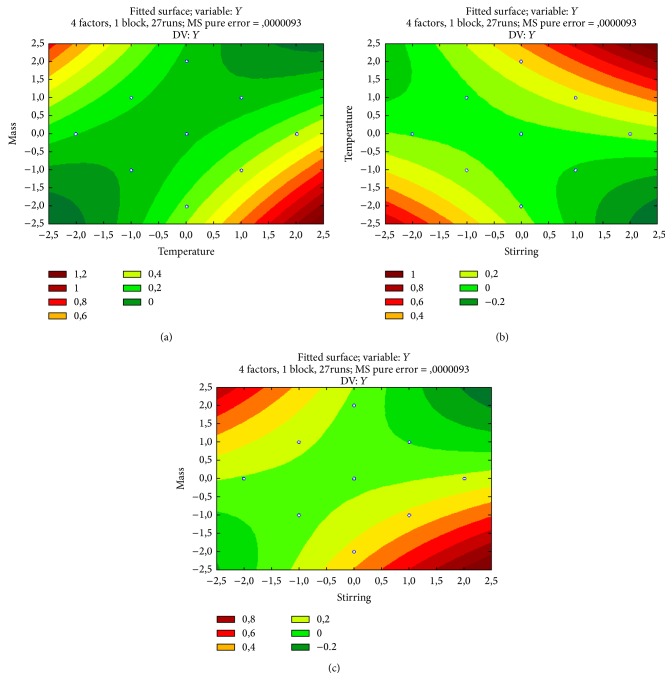
Response surface obtained by the CCRD mathematical model design for the extraction of resveratrol by the microwave-assisted method as a function of (a) temperature and mass; (b) temperature and stirring; and (c) stirring and mass.

Following the first factorial design mentioned previously, a central composite rotatable design (CCRD) was employed in order to obtain the optimum conditions for extracting* trans*-resveratrol. The reaction parameters involved were the same as those described above, with optimization of the intervals studied. Variables, along with their coding and uncoded values, are presented in Table 3. In CCRD, the selected variables were varied in five levels, resulting in 27 trials, including eight axial points and three central points to monitor the curvature. In order to fit a second-order model eight extra points with the same distance from the central point were added to the matrix of this card. The results presented showed that excellent extraction can be obtained by the optimization of reaction conditions affording the desired product (entry 8; Table 3) (Figure 2). In respect to the estimation of the effects by CCRD, it was possible to see (Supplemental Material 1B) that the quadratic effects, which shows the variables temperature, solvent, stirring, and the interaction between each other, were significant in the process (*p* < 0.05). These results are illustrated in Figures 2(a), 2(b), and 2(c). A negative effect of the variable solvent : mass proportion in the range studied is observed, perhaps, due to the low homogeneity of the medium generated by increasing the concentration of the substrate, which in turn undermines the contact of the substrate with the solvent of extracting. The other variables presented positive effects, in contrast to what was observed on the first design performed. It is justified by an agreement of the model on the exact ranges. The experimental data have been adjusted to the proposed model and adequacy was performed by the analysis of variance and parameter *R*^2^ and statistical testing of the model was done by Fisher's statistical test for ANOVA. Equation (1) represents the mathematical model of the extraction of* trans*-resveratrol in function of the variables.

(1) Y=0.073333+0.008500·St+0.008854·St2+0.052750·T+0.33479·T2+0.017167·S+0.036479·S2−0.041917·m+0.020604·m2+0.081875·St·T+0.052750·St·S−0.072250·St·m+0.082500·T·S−0.092750·T·m−0.069·S·m,

where *Y* is the percentage yield extraction and *T*, *S*, St, and *m* are the uncoded values of temperature, solvent ratio, stirring, and solid : solvent ratio, respectively.

Statistical testing of the model was performed by Fisher's statistical test for ANOVA (Supplemental Material 1C). The table represents the analysis of variance (ANOVA) which shows the validity of the model by *F* test and residue that shows the magnitude of experimental error. The calculated *F* (12.32) was higher than the tabulated *F* (2.63), showing the validity of the experimental model. The goodness of the model can be checked by the determination (*R*^2^). The determination coefficient (*R*^2^ = 0.93) implies that the sample variation of 93% for* trans*-resveratrol extraction is attributed to the independent variables and can be accurately explained by the model.  Figure 2(b) shows that as much as the temperature and the stirring are increased, it conducts to optimal* trans*-resveratrol concentration response. Similarly, higher temperatures or stirrings correlated to reduced mass (solid : solvent ratio) produce this same effect (Figures 2(a) and 2(c), resp.).

### 3.4. General Considerations

As can be seen in Table 4* trans*-resveratrol was extracted from all peanut samples investigated and the results showed that higher amounts were extracted by the sonication method (Supplemental Material 1D). This suggested that the sonication method should be more effective in the extraction of* trans*-resveratrol from the leaves of* Arachis repens* compared to MAE and maceration, despite the fact that MAE showed to be an excellent choice since it extracted high yields in a reduced time. This may be because sonication causes a further disruption of the cells, thereby facilitating mass transfer of the cellular components. The efficiency of ultrasound has already been explained in the literature [24–26] as having worked with extracts from other species. These authors have also concluded that ultrasound-assisted methods are faster and furnish yield positive results. The* trans*-resveratrol extraction has already been previously reported in literature, but, in minor amounts, such as in pistachio (0.09–1.67 *μ*g/g), cocoa products (0.4–0.5 *μ*g/g), peanut products (0.06–5.14 *μ*g/g),* Vaccinium* species (0.01–5.88 *μ*g/g dry weight), and grape skins (11.1–123.0 *μ*g/g dry weight) [21–23, 27–30].

## 4. Conclusions

As a conclusion, the best condition for macerating the leaves of* A. repens*, at a laboratory level, was 80% MeOH, 1 h of solvent exposure at 40 mL/g dry material. The antioxidant activity and the* trans*-resveratrol contents increased from 30 to 60 min extraction time. Therefore, both decreased sharply at 240 min, possibly, due to the decomposition of active compounds. The maximum yield of total phenolic compounds,* trans*-resveratrol, and antioxidant activity behavior, observed by the UAE, was in response to ultrasound by 5 min after a maceration pretreatment by 5 min, in the ratio of 1 : 40 (solid-solvent). In addition, we have used the RSM setting as an important tool for optimization extraction of* trans*-resveratrol by MAE. After an initial evaluation by FFD, the CCRD was chosen for further development leading to an increase on extraction from 0.058 mg/L to final 2.516 mg/L at 90% MeOH, 60°C, mass 1 : 35 (w/v), and 1200 rpm for 20 min. The mathematic model proposed suggested a satisfactory representation of the process and good correlation among the experimental results and the theoretical values predicted by the model equation were achieved. Within the parameters evaluated on the present work, the use of UAE, as well as the maceration and MAE, in optimized conditions, showed to be more efficient than the conventional maceration methods previously described for* trans*-resveratrol. Considering that, this species can be considered as a new source of* trans*-resveratrol, an important bioactive compound with important health benefits when consumed.

## Supplementary Material

Supplementary material 1A: Effects estimatives of the independent variables investigated by FFD for the optimyzed microwave-assisted extraction of resveratrol. Supplementary material 1B: Effects estimatives of the independent variables investigated by CCRD for the optimyzed microwave-assisted extraction of resveratrol.

## Figures and Tables

**Table 1:** Total phenolic compounds contents in GAE proceeding from different extracts obtained from the leaves of *A. repens* measured by the Folin-Ciocalteu method.

Entry	Extraction conditions		
	*Maceration* (80% MeOH aq.) (v/v) and 1 : 40 (solid : liquid)	GAE contents on extracts obtained by different times of *maceration* exposure	
		Time (hours)	GAE (500 mg/g)

1		0.5	30.18 ± 1.38^b^
2		1	58.10 ± 1.36^a^
3		2	30.28 ± 1.49^b^
4		3	31.17 ± 0.97^b^

	*Ultrasound* (80% MeOH aq.) (v/v) and 1 : 40 (solid : liquid)	GAE contents on extracts obtained by different times of *ultrasound* exposure	
		Time (minutes)	GAE (500 mg/g)

5		5	212.51 ± 2.22^a^
6		10	185.82 ± 3.06^b^
7		20	144.77 ± 0.22^c^
8		30	140.82 ± 1.13^d^

	*Maceration + ultrasound (5 min)* (80% MeOH aq.) (v/v) and 1 : 40 (solid : liquid)	GAE contents on extracts obtained by different times of *maceration* exposure	
		Time (minutes)	GAE (500 mg/g)

9		5	357.18 ± 1.83^a^
10		10	189.47 ± 2.95^b^
11		20	136.50 ± 1.01^c^
12		30	104.14 ± 1.52^d^

	*Maceration (5 min) + ultrasound (5 min)* (80% MeOH aq.) (v/v) in different ratios (solid-solvent) (g/mL)	GAE contents of extracts obtained by different ratios (solid-solvent) of *maceration* and *ultrasound *exposure	
		Relation (solid-solvent)	GAE (500 mg/g)

13		1 : 20	246.83 ± 2.48^e^
14		1 : 30	315.51 ± 0.84^b^
15		1 : 40	356.17 ± 1.36^a^
16		1 : 50	299.61 ± 1.14^c^

Means ± standard error followed by the same letter within each column are not significantly different at *p* = 0.05, Tukey-Kramer test.

**Table 2:** Fractional factorial design (FFD) model totalizing 11 experiments aiming at the optimization of *trans*-resveratrol extraction in mg/g extract, by the microwave-assisted technique.

Entry	St (rpm)	*T* (°C)	Solvent concentration (MeOH aq.) (%)	Mass : solvent proportion (g/mL)	Content of *t-*resveratrol (mg/g extract)^a^
1	−1 (600)	−1 (30)	−1 (60)	−1 (1 : 60)	0.235
2	1 (1200)	−1 (30)	−1 (60)	1 (1 : 20)	0.786
3	−1 (600)	1 (70)	−1 (60)	1 (1 : 20)	0.075
4	1 (1200)	1 (70)	−1 (60)	−1 (1 : 60)	0.456
5	−1 (600)	−1 (30)	1 (100)	1 (1 : 20)	0.231
6	1 (1200)	−1 (30)	1 (100)	−1 (1 : 60)	0.085
7	−1 (600)	1 (70)	1 (100)	−1 (1 : 60)	0.103
8	1 (1200)	1 (70)	1 (100)	1 (1 : 20)	0.289
9	0 (900)	0 (50)	0 (80)	0 (1 : 40)	0.143
10	0 (900)	0 (50)	0 (80)	0 (1 : 40)	0.117
11	0 (900)	0 (50)	0 (80)	0 (1 : 40)	0.124

^a^Measured by HPLC-DAD.

**Table 3:** Real and coded values (+ level, 0 intermediate, − lower level) for the independent variables, 2^4–1^ experimental factorial design and results of CCRD for resveratrol extraction by the microwave-assisted technique.

Entry	Variable levels				*t*-resveratrol (mg/g extract)^a^
	Stirring (rpm)	Temperature (°C)	Solvent conc. (MeOH aq.) (%)	Solvent : mass proportion (g/mL)	
1	−1 (975)	−1 (37.5)	−1 (75)	−1 (1 : 30)	0.149
2	1 (1125)	−1 (37.5)	−1 (75)	−1 (1 : 30)	0.062
3	−1 (975)	1 (52.5)	−1 (75)	−1 (1 : 30)	0.064
4	1 (1125)	1 (52.5)	−1 (75)	−1 (1 : 30)	0.343
5	−1 (975)	−1 (37.5)	1 (85)	−1 (1 : 30)	0.065
6	1 (1125)	−1 (37.5)	1 (85)	−1 (1 : 30)	0.071
7	−1 (975)	1 (52.5)	1 (85)	−1 (1 : 30)	0.302
8	1 (1125)	1 (52.5)	1 (85)	−1 (1 : 30)	0.809
9	−1 (975)	−1 (37.5)	−1 (75)	1 (1 : 20)	0.527
10	1 (1125)	−1 (37.5)	−1 (75)	1 (1 : 20)	0.090
11	−1 (975)	1 (52.5)	−1 (75)	1 (1 : 20)	0.120
12	1 (1125)	1 (52.5)	−1 (75)	1 (1 : 20)	0.070
13	−1 (975)	−1 (37.5)	1 (85)	1 (1 : 20)	0.058
14	1 (1125)	−1 (37.5)	1 (85)	1 (1 : 20)	0.048
15	−1 (975)	1 (52.5)	1 (85)	1 (1 : 20)	0.087
16	1 (1125)	1 (52.5)	1 (85)	1 (1 : 20)	0.133
17	−2 (1200)	0 (45)	0 (80)	0 (1 : 25)	0.092
18	−2 (1200)	0 (45)	0 (80)	0 (1 : 25)	0.067
19	0 (1050)	−2 (30)	0 (80)	0 (1 : 25)	0.076
20	0 (1050)	−2 (30)	0 (80)	0 (1 : 25)	0.280
21	0 (1050)	0 (45)	−2 (90)	0 (1 : 25)	0.124
22	0 (1050)	0 (45)	−2 (90)	0 (1 : 25)	0.256
23	0 (1050)	0 (45)	0 (80)	−2 (1 : 35)	0.195
24	0 (1050)	0 (45)	0 (80)	−2 (1 : 35)	0.058
25	0 (1050)	0 (45)	0 (80)	0 (1 : 25)	0.074
26	0 (1050)	0 (45)	0 (80)	0 (1 : 25)	0.070
27	0 (1050)	0 (45)	0 (80)	0 (1 : 25)	0.076

^a^Measured by HPLC-DAD based on a calibration curve of *trans-*resveratrol in five different levels.

**Table 4:** * Trans*-resveratrol contents (mg/g extract) obtained in the present study by different extractive methodologies in optimized conditions.

Methodology	Time	Methanol %	Stirring (rpm)	Temperature (°C)	Relation solid-solvent (w/v)	*t-*resveratrol content (mg/g extract)
Maceration	1 hour	80	—	—	1 : 50	1.024 ± 0.036
Ultrasound assisted	5 minutes	80	—	—	1 : 40	3.959 ± 0.675
Microwave assisted	20 minutes	90	1200	60	1 : 35	2.516
