# Supplementary material for: Optimized Extraction of Resveratrol from Arachis repens Handro by Ultrasound and Microwave: A Correlation Study with the Antioxidant Properties and Phenol Contents
Source: ScientificWorldJournal. 2016 Dec 27;2016:5890897. doi: 10.1155/2016/5890897 (PMC5223070; doi:10.1155/2016/5890897)
Supplement: Supplementary file 1 — Supplementary material 1A: Effects estimatives of the independent variables investigated by FFD for the optimyzed microwave-assisted extraction of resveratrol. Supplementary material 1B: Effects estimatives of the independent variables investigated by CCRD for the optimyzed microwave-assisted extraction of resveratrol. [file 5890897.f1.docx]

**Supplemental Material 1A**

Estimating effects of the independent variables investigated by FFD for the optimization of microwave assisted extraction of resveratrol.


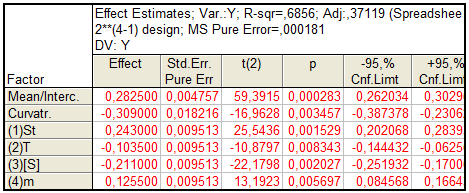


**Suplemental Material 1B**

Estimating effects of the independent variables investigated by CCRD for the optimization of microwave assisted extraction of resveratrol.

**Suplemental Material 1C**

**Table** – Variance analysis for validation of the mathematical models (ANOVA) in CCRD.

| Factor | Sum of square | Degrees of freedom | Mean square | *F* calculated | *F* tabulated | *p-*Value |
| --- | --- | --- | --- | --- | --- | --- |
| Regression | 0.720000 | 14 | 0.051317 | 12.32923 | 2.637124 | 4.9469E-05 |
| Residuals | 0.050000 | 12 | 0.004162 | 0.833645 |  |  |
| Lack of fit | 0.049928 | 10 | 0.004993 | 534.9393 |  |  |
| Pure error | 0.000019 | 2 | .,33E-06 |  |  |  |
| TOTAL | 0.768380 | 26 |  |  |  |  |

^a^ Confidence level 95%.

**Suplemental Material 1D**


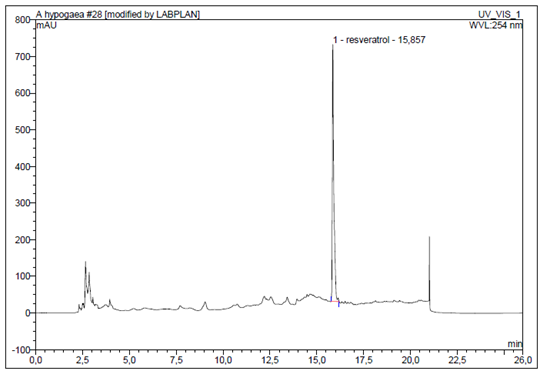


**Figure 1D(a)**. Chromatogram of the *trans-*resveratrol (RSV) Standard used in this work.


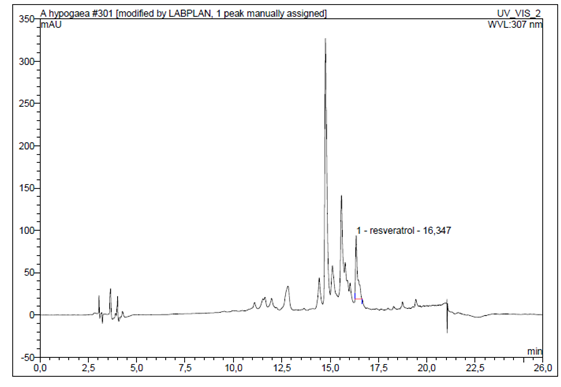


**Figure 1D(b)**. Chromatogram of the sample obtained by the ultrasound extraction technique.
